# The laparoscopically harvested omental flap and transverse plate fixation for sternal reconstruction in complicated sternal wound infection after cardiac surgery

**DOI:** 10.1186/1749-8090-8-S1-P58

**Published:** 2013-09-11

**Authors:** JM De Raet, PT Sergeant

**Affiliations:** 1 Department of Cardiac Surgery, Heart Center Leipzig, University of Leipzig, Leipzig, Germany; 2 Department of Cardiac Surgery, University Hospitals Leuven, Leuven, Belgium

## Background

Complicated sternal wound infection after cardiac surgery has an incidence of 0.4 – 6.9 % and mortality of 7 – 80 %. The ideal reconstructive procedure is still a matter of debate.

## Aim

To report our experience with the laparoscopically harvested omental flap and transverse plate fixation for sternal reconstruction after complicated sternal wound infection.

## Methods

Between 2010 and 2011, 6 patients with type IV deep sternal wound infection underwent a sternal reconstruction with a laparoscopically harvested omental flap and transverse plate fixation. The median age of the cohort (1 female and 5 males), was 72.5 years (range: 49-78 years). In 5 patients, a bilateral internal thoracic artery had been used. Considerable preoperative risk factors were present: Obesity with Body Mass Index (BMI) ≥ 33 (range: 33 – 35: 3 patients); chronic obstructive pulmonary disease (COPD) without steroid therapy preoperatively (4 patients); Diabetes mellitus (type 1: 2 patients; type 2: 1 patient). Abdominal surgery had previously been performed in 4 patients. In 5 cases, the mediastinal wound was prepared with negative pressure wound therapy following surgical debridement. An internal fixation of the sternum was made by titanium locking plates with sternal and rib-to-rib fixation. The postoperative course of these patients was followed by clinical follow-up.

## Results

Early postoperative sternal stability was seen in all 6 patients. The 30-day perioperative mortality rate was zero, with an overall survival of 100% until today. Postoperatively no superficial or deep surgical site infections (SSI) were appreciated. Follow-up ranged from 24 to 41 months (median: 28 months).

## Conclusions

Combination of a laparoscopically harvested omental flap and transverse plate fixation can contribute to a successful outcome following complicated sternal wound Infection and deserves serious consideration, regardless of the co-morbidity or previous abdominal surgery.

